# Lived experiences of Ugandan women who had recovered from a clinical diagnosis of postpartum depression: a phenomenological study

**DOI:** 10.1186/s12884-021-04287-2

**Published:** 2021-12-13

**Authors:** Catherine Atuhaire, Godfrey Zari Rukundo, Laura Brennaman, Samuel Nambile Cumber, Grace Nambozi

**Affiliations:** Faculty of Medicine, Department of Nursing, Mbarara University of Science and Technology (MUST), Mbarara, Uganda

**Keywords:** Postpartum depression, Lived experiences, Uganda, Qualitative research

## Abstract

**Background:**

Postpartum depression affects a significant proportion of women of childbearing age. The birth of a newborn baby is normally considered a joyful event, inhibiting mothers from expressing their depressive feelings. If the condition is not well understood and managed, mothers with postpartum depression are likely to experience suicidal ideation or even commit suicide. This study explored lived experiences of women who had recovered from a clinical diagnosis of postpartum depression in southwestern Uganda.

**Methods:**

This phenomenological study adopted the explorative approach through in-depth interviews as guided by the biopsychosocial model of depression. It was conducted in Mbarara Regional Referral Hospital, Bwizibwera Health Centre IV and Kinoni Health Centre IV located in Mbarara and Rwampara districts, southwestern Uganda. Data were collected from 30 postpartum mothers who were purposively selected, between 9th December 2019 and 25th September 2020. We analyzed this work using thematic data analysis and this was steered by the Colaizzi’s six-step phenomenological approach of inquiry.

**Results:**

The findings were summarized into five major themes: 1) somatic experiences including insomnia and headache, breast pain, poor breast milk production, weight loss and lack of energy; 2) difficulties in home and family life including overwhelming domestic chores, lack of social support from other family members, fighting at home and financial constraints due to COVID-19 pandemic; 3) negative emotions including anger, self-blame, despondency and feelings of loneliness and regrets of conceiving or marriage; 4) feelings of suicide, homicide and self-harm including suicidal ideation and attempt, homicidal ideations and attempt and feelings of self-harm and 5) coping with postpartum depression including spirituality, termination of or attempt to leave their marital relationships, acceptance, counselling and seeking medical treatment, perseverance.

**Conclusion and recommendations:**

Suicidal and homicidal thoughts are important parts of the postpartum depression experience, and these may put the lives of the mothers, their spouses and their babies at a great risk. Poor relationship quality, intimate partner violence and lack of financial resources contribute significantly to the negative emotional experiences of mothers with PPD.

**Supplementary Information:**

The online version contains supplementary material available at 10.1186/s12884-021-04287-2.

## Background to the study

Depression is one of the most treatable mental illnesses and the second leading cause of global burden of disease [1]. Women are twice as likely to be diagnosed with depression as compared to men [2, 3]. In addition to the general burden of depression, women of childbearing age also suffer from postpartum depression (PPD) following childbirth [4]. Although PPD may have its onset prenatally, this study focuses on PPD that may be experienced up to 6 weeks following childbirth or may take 10 weeks before a mother acknowledges these depressive feelings [5]. In most circumstances, the birth of a newborn baby is normally considered a joyful experience and mothers may therefore be embarrassed to express their depressive feelings during this period [6]. PPD has potential negative consequences to the psychosocial and economic status of the mothers, their spouses, their newborn babies and their relatives. Without adequate recognition, treatment and support, some women experiencing severe PPD are at greater risk of suicidal behavior, homicide or self-harm [7–9].

Decades of interdisciplinary research have produced a significant body of knowledge about PPD such as measurement scales, diagnosis, predictors, characteristics, treatment and consequences of untreated PPD [10, 11]. However, the existing research is primarily from high income countries and does not explore lived experiences of women with PPD in sub-Saharan Africa (SSA) and other low-income settings. Lived experiences generally refer to the presentations of the experiences; choices and options of a given person and how such factors influence one’s knowledge, perceptions and actions [12, 13]. Prior studies have reported a wide range of experiences with potential adverse effects on maternal, partner and infant well-being [14, 15]. Lived experiences described in previous studies include feeling misunderstood and alienated, perceived lack of competence as mothers, feelings of anxiety, loneliness, feeling overwhelmed, feelings of guilt, social avoidance, adjustment difficulties, unwelcome emotions, breastfeeding difficulties, self-blame, and withdrawal symptoms [15, 16]. Nearly all the studies that Maxwell and colleagues included in their meta-analysis were comprised of samples from high-income countries and they reported lack of interest and bonding with the new baby, anxiety about the baby, appetite loss, feelings of being a bad mother, fear of harming oneself or one’s baby, crying, hopelessness, worthlessness, and sadness as common experiences of women diagnosed with PPD [17].

A recent Ugandan study reported a PPD prevalence of 27.1% among 6 weeks postpartum mothers, but the experiences these mothers go through and how they cope with PPD is unknown [18]. A deeper understanding of what this phenomenon means from a mother’s perspective can inform policy and development of appropriate interventions against PPD. In this current study we explored the lived experiences of women who had recovered from a clinical diagnosis of PPD in Mbarara and Rwampara districts in southwestern Uganda.

## Methods

### Study design

This phenomenological study adopted the explorative approach to qualitative inquiry. The phenomenon was postpartum depression and the perspective was nursing science.

### Study setting

This study was conducted in Mbarara Regional Referral Hospital (MRRH), Bwizibwera Health Centre IV (BHC IV) and Kinoni Health Centre IV (KHC IV), all located in Mbarara and Rwampara districts, southwestern, and they are approximately 270 km from Kampala (capital city of Uganda). MRRH is in the urban setting and the biggest regional referral hospital in southwestern Uganda with in-patient and out-patient services. BHC IV and KHC IV are in the peri-urban setting but they serve mothers from the rural parts of Mbarara and Rwampara districts. All these public health facilities offer maternal-child health services.

### Study population and period

Data were collected between 9th December 2019 and 25th October 2020 from mothers who were from ten-weeks to 18 weeks’ postpartum. This period was selected to take advantage of the participants’ full experiences and to allow them to reflect on the meaning of their lived experiences.

### Inclusion and exclusion criteria

Mothers were considered eligible if they had been clinically diagnosed according to the Diagnostic and Statistical Manual V (DSM-5) criteria with PPD at 6 weeks postpartum in a preliminary quantitative study that was earlier carried out, be depression free following treatment and they were at least 10 weeks’ post-partum. In addition, all eligible mothers had to be above 18 years of age and they had delivered live births to take part in the study. While recruiting these participants, the interviewer screened potential participants using the DSM-5 to ensure that the PPD had recovered prior to the interview. Participants were ineligible if the screening revealing PPD remained present. These deferred participants were referred to the study psychiatrist for further management and excluded from the study.

### Sample size estimation and sampling procedure

Purposive sampling was used to explore the lived experiences of mothers who were diagnosed with postpartum depression. Sampling and data collection continued to the point where no new information was obtained [19]. Saturation was achieved at 30 respondents.

### Data collection tools

A semi-structured interview guide with open ended questions was developed by the authors guided by the study aims, the biopsychosocial model of depression and used for data collection. Open-ended questions stimulated the interviewee to relate freely any thoughts she had. An interview guide was used to conduct the interviews and explored various experiences of the participants including personal, psycho-social, economic, biological experiences and relationships with their children, spouses, close relatives, and communities at large. The guide included exploring the participants’ coping strategies. See Additional file 1.

### Data collection procedure

The researchers identified 38 potential participants as meeting inclusion criteria from the quantitative prevalence arm of the study project and contacted them by phone. Out of the 38 participants, 30 accepted to participate in the study. In-depth interviews (IDIs) with mothers who were eligible for this study were conducted in either Runyankore-Rukiga or English as preferred by the participant. These interviews occurred in a private room or quiet open space for adequate privacy at the health facilities. All in-depth interviews were audio-recorded. Each interview lasted 30 to 45 min. There was a moderator and a note taker for all the in-depth interviews to supplement the audio recordings. The first author and a research assistant collected the data.

Probing questions were also used without interrupting the flow of the interviews to get more clarification and answers for additional aspects of their experiences. The interview guide was first piloted with women who met study criteria from Municipal Health Centre IV, but not included in the study analysis and there were no modifications made.

### Data analysis

All the IDIs were transcribed verbatim. The transcripts in the Runyankore-Rukiga language were translated to English by a language expert. The first author thoroughly checked the translations from Runyankore-Rukiga to English to reduce the risk of misinterpretation of the data. All transcribed audio-recorded interviews were cross checked with the recordings to ensure accuracy. Thematic analysis, as described by Braun and Clarke was the appropriate framework for analysis due to the explorative nature and the study aim [20]. The steps in the thematic analysis were steered by the Colaizzi’s six-step phenomenological approach to enquiry [20]. In the first step, CA familiarized herself with the data, by reading and re-reading all the transcribed interviews. All statements were extracted in the accounts that were of direct relevance to the phenomenon under investigation (initial coding process) in the second step. In the third step where she identified the broader themes present in the set of codes relevant to the phenomenon that arose from a careful consideration of the significant statements. During this process, pertinent quotes were broadly categorized, then, themes were generated based on multiple statements that conveyed similar meanings. Atlas.ti version 8 software was used electronically to sort and organize the large dataset. The researcher reflexively “bracketed” her pre-suppositions to stick closely to the phenomenon as experienced by the respondents. In the fourth step, CA reflected and systematized the codes into larger units. Steps 1 to 3 were repeated for each interview and identified meanings were clustered into themes that were common across all accounts. An inductive approach was used to find the correlation between codes, sub-themes, themes, and the framework as seen in the coding framework in Table 1. The coding framework coincided with the bio-psychosocial model of depression. Themes were defined and named in the fifth step and finally a thematic analysis report was written. This iterative process was continuously guided by the co-authors.

**Table 1:** Illustration of the data analysis process

Verbatim	Codes	Sub themes	Themes
*“I used to get sleepless nights. I would spend the whole night awake. Whenever it would get to 2.00 am, I would sit in bed with no sleep at all because of this stressful relationship that was lacking the basic needs (B 25).”*	Disturbed sleep	Insomnia & headache	Somatic experiences
*“After delivering my breast started swelling, I tried using herbal medicine but all in vain and I decided to go to … to seek treatment but instead I was told to go to … Unfortunately, I didn’t have enough money, instead I decided to get some local treatment by burning the boil at the traditional healer but the wound from the burns have never healed until now … Right now I use only one breast to feed my child and also buy milk because milk from one breast is always not enough. So this is a very big challenge to me and my baby because she gets poor feeding and she is always crying (KHCIV 11).”*	Pain	Breast pain	
*“I think lack of breast milk disturbed me, because when I lost weight due to lack of appetite and as a result, my breast milk reduced… it was challenging to feed the baby which maybe… I think it is because of weight loss and lack of energy (BHCIV 27).”*	Lack of breast milk	Poor milk production	
*“Okay I lost weight after losing appetite because I was not feeding well … at the health facility they asked me what happened that I reduced weight, my kilograms have reduced. I reduced weight, count from 97kgs to 65kgs due to too much stress (MRRH 6).”*	Losing weight	Weight loss	
*“… after delivering I bled so much and the blood would just flow. It was not an easy experience since I was feeling very tired and weak due to losing a lot of blood and sometimes I failed to eat or had no food to eat and drink as the doctors had advised leading to dizziness ... (KHCIV 12).”*	Fatigue	Lack of energy	
“*Otherwise I do most of the work like washing, cooking, sweeping, mopping myself and when I feel tired and sometimes fail to eat … Yes, there is when I think that maybe I leave or that I sleep the whole day but the baby’s clothes need to be washed yet my husband can never support me… not even to hold the baby as I do them (MRRH 04).”*	Not assisted with house chores besides the new baby roles	Overwhelming domestic chores	Difficulties in home and family life
*“My husband lost his first wife leaving him with five children. I have given him three more children. My in-laws are just full of hate, they complain about me not taking good care of the children and all this just increases my stress and high blood pressure (KHCIV 15).”*	Deficient family social support	Lack of social support from other family members	
*“When my husband drinks, he starts quarrelling from say 10:00 pm until morning without sleeping … but as a man, he cannot fail to give you some slaps (BHCIV 33).”*	Conflict with partner	Fighting at home	
*“I stopped working because I got challenges with my spinal cord. Right now, it is difficult to support myself and my children. My husband does not support us in any way. This is driving me crazy (KHCIV 11).”*	Income challenges	Financial constraints during COVID-19 pandemic	

### Rigor of this study

Guba’s model of trustworthiness was employed [21]. A naturalistic paradigm of trustworthiness inclusive of four aspects: credibility, transferability, conformability, and dependability that underpinned the approaches undertaken throughout the study [19]. Credibility was ensured through establishing a conducive rapport with participants and telling their story and unique experience through the Collaizi method of data analysis that ensured accuracy of the findings. Conformability was done by evaluating interview questions to ensure that they were open ended and not leading. Codes used for each respondent were allowed for proper matching of each respondent’s description. A clear trail was created where all steps taken in the research were outlined and made available to members of the research team for scrutiny. Dependability was achieved by accurately documenting the processes undertaken. This detailed documentation will enable the readers to ascertain that appropriate research methods had been adhered to and provide future researchers with the depth of information needed to replicate the study in the reader’s context [22]. Finally, transferability was achieved by eliciting thick descriptions of study methods and findings, adequate sampling and achieving data saturation. It was also supported because participants were selected purposively [21].

## Results

### Characteristics of study participants

The interviews of participants from two health centres (Bwizibwera and Kinoni Health Centre IV) and one regional referral hospital in south-western Uganda were analysed. The 30 participants in the study were females between 18 and 37 years old with mean age of 28 years. Most of them were married (73%), had attended up to primary level of education (40%), were unemployed (53%) and had two children (37%). See Table 2.

**Table 2:** Participants’ Characteristics *N* = 30

Variables	Frequency
Health Facility	
Bwizibwera HCIV	12
Kinoni HCIV	6
Mbarara Regional Referral Hospital	12
Age (range 18–37 years)	
18–20	3
21–29	15
30–37	12
Marital status	
Married	22
Unmarried	8
Level of education	
No education	4
Primary school	12
Secondary school	7
Tertiary level/ college	7
Employment status	
Employed	14
Unemployed	16
Number of children	
One	6
Two	11
Three	7
Four or more	6

Five major themes emerged from the data: 1) somatic experiences; 2) difficulties in home and family life; 3) negative emotions; 4) feelings of suicide, homicide and self-harm and 5) coping with postpartum depression by engaging in diversional activities. These themes and their sub-themes are presented in this section supported by participants’ verbatim comments as presented in italics. See Table 3 below:

**Table 3:** Generated themes and sub themes

Generic Themes	Sub Themes
Somatic experiences	Insomnia and headache
	Breast pain
	Poor milk production
	Weight loss
	Lack of energy
Difficulties in home and family life	Overwhelming domestic chores
	Lack of social support from other family members
	Fighting at home
	Financial constraints during COVID-19 pandemic
Negative emotions	Anger
	Self-blame
	Despondency and feelings of loneliness
	Regrets of conceiving or marriage
Feelings of suicidal, homicide and self-harm	Suicidal ideations and attempt
	Homicidal ideations and attempt
	Feelings of self-harm
Coping with Postpartum depression	Spirituality: (Christians, Muslims and Traditional African beliefs)
	Terminating or attempting to leave their marital relationships
	Acceptance, counselling and seeking medical treatment
	Perseverance

#### Somatic experiences

Six sub themes emerged from somatic experiences that the participants attributed to the distress they were going through hence postpartum depression: insomnia and headache, breast pain, poor breast milk production, weight loss and lack of energy:

##### Insomnia and headache

Some mothers in the study reported sleep challenges that was characterized by interruptions in sleep patterns and sleepless nights during the postpartum period as expressed by participant KHCIV 13. BHCIV 25 and others reported that they were suffering from unexplained headaches. One of the mothers narrated that she visited the health facility due to the headache:

*“Immediately I fell so sick after I delivered my child, I developed too much headache, pain in the legs and developed much stress. I tried to take some medication but all in vain … they went ahead and tested me for malaria but the results showed a negative position (KHCIV 13).”*

*“I used to get sleepless nights. I would spend the whole night awake. Whenever it would get to 2.00 am, I would sit in bed with no sleep at all because of this stressful relationship that was lacking the basic needs (BHCIV 25).”*

##### Breast pain

A mother reported that one of her breasts had severe pain and swelling. She was referred to the referral hospital, but she failed to make the appointment due to the financial constraints and therefore resorted to seeking treatment from a local traditional healer who burnt the breast. At the time of data collection, she was still struggling with the burns. She expressed that she was using one breast to feed her baby, though the milk production was not enough and this stimulated stress. The failure for the baby to receive sufficient breast milk worsened her stress:

*“After delivering my breast started swelling, I tried using herbal medicine but all in vain and I decided to go to … to seek treatment but instead I was told to go to … Unfortunately, I didn’t have enough money, instead I decided to get some local treatment by burning the boil at the traditional healer but the wound from the burns have never healed until now… Right now, I use only one breast to feed my child and also buy milk because milk from one breast is always not enough. So this is a very big challenge to me and my baby because she gets poor feeding and she is always crying (KHCIV 11).”*

##### Poor breast milk production

On the other hand, some mothers described the challenges surrounding breastfeeding of poor milk production due to decreased appetite that was triggered by stress. They reported experiencing poor or no breast milk production:

*“I think lack of breast milk disturbed me, because when I lost weight due to lack of appetite and as a result, my breast milk reduced… it was challenging to feed the baby which maybe… I think it is because of weight loss and lack of energy (BHCIV 27).”*

##### Weight loss

Some of the mothers recounted that they had lost appetite which led to weight reduction to an extent that some of them were not happy with their body image as narrated by participant MRRH 6:

*“Okay I lost weight after losing appetite because I was not feeding well … at the health facility they asked me what happened that I reduced weight, my kilograms have reduced. I reduced weight, count from 97kgs to 65kgs due to too much stress (MRRH 6).”*

##### Lack of energy

Mothers also related the lack of energy to poor eating habits that led to them feeling dizzy to an extent that they could not cultivate land, which is their source of income, or even eat which worsens their physical state. Some mothers said they lacked energy and felt dizzy which was related to the blood loss that they had suffered after delivery:

*“… after delivering I bled so much and the blood would just flow. It was not an easy experience since I was feeling very tired and weak due to losing a lot of blood and sometimes, I failed to eat or had no food to eat and drink as the doctors had advised leading to dizziness ... (KHCIV 12).”*

#### Difficulties in home and family life

Four sub themes under this theme came from the participants’ reports. These included overwhelming domestic chores, lack of social support from other family members, fighting at home and financial constraints due to COVID-19 pandemic:

##### Overwhelming domestic chores

Mothers also revealed that childbirth had added extra chores to the common tasks usually performed by mothers such as cooking, sweeping, cleaning utensils, washing clothes and taking care of other children. Such tasks are performed in addition to childbirth-related tasks like breastfeeding, washing the baby’s clothes and attending to the infants when they cry. Participants revealed that such chores often leave them exhausted and tired, most especially when their partners do not support them with some of these tasks while they rest:

“*Otherwise I do most of the work like washing, cooking, sweeping, mopping myself and when I feel tired and sometimes fail to eat … Yes, there is when I think that maybe I leave or that I sleep the whole day, but the baby’s clothes need to be washed. Yet my husband can never support me… not even to hold the baby as I do them (MRRH 04).”*

##### Lack of social support from other family members

Most mothers express the need for social support from close family members. This could be emotional or tangible support like assistance with the baby and accompanying the mother to the health facility for delivery. However, most of the women reported negative experiences and lack of social support from family members. These mothers also reported limited support from their other social circles besides family:

*“My husband lost his first wife leaving him with five children. I have given him three more children. My in-laws are just full of hate, they complain about me not taking good care of the children and all this just increases my stress and high blood pressure (KHCIV 15).”*

##### Fighting at home

Verbal, emotional and sometimes physical abuse were commonly reported by most participants. In some instances, violence was reported if the partner abused alcohol:

*“I am already suffering enough, my husband is an alcoholic and when he drinks, he fights, the whole home becomes messed up, whatever he gets hold of he uses it to beat me up, he breaks cups, plates and anything in his way, this really stresses me further (KHCIV 13).”*

*“When my husband drinks, he starts quarrelling from say 10:00pm until morning without sleeping … but as a man, he cannot fail to give you some slaps (BHCIV 33).”*

##### Financial constraints during COVID-19 pandemic

The majority of participants reported financial and other logistical constraints like food and housing insecurity and lacking clothing for their children. Some of the women lost their jobs or source of income due to COVID-19. This had a significant impact on their emotional and psychological state. The majority of women mainly depended on their partners for their financial and other material needs, yet these described these as unmet needs which may have made them vulnerable to PPD:

*“I stopped working because I got challenges with my spinal cord. Right now, it is difficult to support myself and my children. My husband does not support us in any way. This is driving me crazy (KHCIV 11).”*

*“Since then, it has been difficult to find another job. I solely depend on my husband who has failed to provide us with basic needs like milk, clothes, food and even paying the house rent…All this just stresses me out (BHCIV 02).”*

*“I am not working and unfortunately my husband who was a taxi driver lost his job during this COVID-19 lockdown…It has been difficult for me and my three months old baby to feed. Worst of it, the landlord has been trying to kick us out for not paying his rent. Recently, my husband asked me to return to my parents because he can no longer pay the rent nor provide food for us. I cried for this but had no solution to the situation (BHCIV 05).”*

#### Negative emotions

Under theme of negative emotions, four sub themes emerged as reported by mothers suffering from PPD. These included anger, self-blame, despondency and feelings of loneliness and regrets of conceiving or marriage:

##### Anger

Some participants recalled that their postpartum period was characterized by rage like anger. One of the mothers tried to describe anger as:

*“….. when I get angry, at that time it’s like ants have entered into my head, it’s like I have entered into a state of madness…. after becoming drunk it’s better for him to at least enter the bed and sleep instead of stressing me by physically attacking me, this situation makes me so angry, do you know this anger that does not allow you to even swallow any food… (MRRH 13).”*

Participant MRRH 12 also narrated that anger during this period had an impact on their loss of appetite, inability to eat hence weight loss whereas participant KHCIV 15 described how the type of anger in the postpartum period made her feel so unhappy to an extent of crying to sleep by herself. It was noted that reduced breast milk required supplementing breast feeding with alternate milk:

*“….. I would become so angry and lack the appetite to even eat…. I cry because if I don’t cry I cannot be okay because I can get so angry because of how I was neglected by my husband and even reduce in size yet I should be breastfeeding (MRRH 12).”*

*“Yes it has affected me because I do not have enough breast milk for my child who is still breast feeding and this has forced me to start putting my child on bottle feeding. She is just six months old, it’s not healthy for her… Honestly I feel so bad and stressed whenever I see my child drinking milk instead of breast milk at such an early age. It just worsens the stress that I already have, I always find myself crying until I sleep (KHCIV 15).”*

##### Self-blame

As a result of these range of emotions, some mothers often attempted to justify their partner’s behavior by blaming themselves. For example, delivering a different gender from what the partner expected:

*“... when I got pregnant, he told me that he wanted a baby girl but for me instead I gave birth to a baby boy. Eh... I think that could be the reason why and I think that it is my fault (MRRH 01).”*

##### Despondency and feelings of loneliness

Some participants reported being in a lonely and stressful partnerships. Some mothers also narrated that their partners would not be fully available to attend to their basic needs. As a result, they expressed feelings of loneliness and abandonment by their partners before, during and after childbirth:

*“After delivery, I was abandoned in the hospital by my husband. It made me so angry… I got annoyed after he rejected me because I thought he was going to be so excited that I have delivered and then come to collect me but instead he did not come and up to now, he has not seen the baby (MRRH15).”*

*“My relationship is very stressful…I can’t sleep in my baby’s father’s house because he is married. He rarely visits us and when I request him to see him, he says that I will bring him trouble. The relationship has been very lonely and stressful but I cannot leave him because I really love him (MRRH12).”*

##### Regrets about conceiving or about marriage

In addition to the above sub-themes, the majority of the mothers developed feelings of regret about having conceived the child and wished they had terminated the pregnancy. One further narrated that she even attempted to abort while she was pregnant by using herbal medicine in vain and some mothers also reported having regrets towards the entire marriage as declared:

*“I regretted getting pregnant in the first place, I even tried talking to a health worker, she told me if I tried removing this pregnancy, I might not make back alive, I tried taking some herbal medicine so that I can lose the pregnancy, but it refused (BHCIV 03).”*

*“I wish I would not have been married to this man so quickly without knowing much about his bad behavior of rejection and poorly treated (MRRH 10).”*

#### Feelings of suicide, homicide and self-harm

Participants revealed a wide range of experiences during the postpartum period which included suicidal ideation and attempt, homicidal ideations and attempt and feelings of self-harm as narrated in the following sub-themes.

##### Suicidal ideation and attempt

Several participants revealed having suicidal thoughts during their postpartum period. Some participants had suicidal thoughts but did not attempt to kill themselves, but others made actual attempts. In recounting their experiences with suicidality, participant BHCIV 33, BHCIV 02, KHCIV 15 and BHCIV 11 narrated that:

*“I have ever thought of killing myself and it was because of the man who stressed me after infecting me, when I discovered after giving birth that I was HIV infected I wanted to commit suicide (BHCIV 33).”*

*“At first, I actually used to wish I could get like an accident and pass away (BHCIV 02).”*

Some of the mothers revealed a detailed plan to kill themselves by using several approaches but they were stopped by the thought of leaving their innocent children behind as narrated:

*“I don’t know what to do about my condition, sometimes I even think of leaving my children behind and committing suicide by drowning myself in a river. There is a day I went to the river and stood there and I was about to do it. I thought about my children and wondered what kind of parent I would be if I did that to my children, they are innocent and they surely don’t deserve it (KHCIV 15).”*

Another mother reported attempts to commit suicide by hanging and later by swallowing pills. However, she eventually abandoned her children and is currently in a new relationship:

*“… when I discovered that I was HIV positive when I was pregnant, I suffered so much that after giving birth, I even tried to commit suicide by hanging myself, I had already put the rope around my neck… but someone found me and cut down the rope so first before it was too late … I did not give up, I tried the second time by swallowing a lot of tablets still I did not die… I even took an excess dose of ARVs but I did not die, actually I vomited everything that I had swallowed and still did not perish, this time I realized that the Lord was saving me for something (BHCIV 11).”*

##### Homicidal ideations and attempt

Some participants described thoughts of killing other people like their children, their partners or friends as narrated:

*“I love myself so much, but I had ever thought of poisoning my husband to death and I leave alone with my children … when the situation was so bad, I would wish him death... I used to think that the widows, sometimes live a peaceful life without being emotionally traumatized by their husbands like mine did. I prayed to God that how I wish he would take him away from this world and I live my life alone (BHCIV 01).”*

*“I develop ideas of doing something bad to my husband… I am like should I get a machete or broad, bladed African knife and cut someone… (MRRH 13).”*

Indeed, one participant revealed that she had planned on killing her children by giving them poison, but she believed that the God she prayed to, saved her when she made the attempt. In fact, she further reported that when her friend tried to stop her, she beat her and tried to get a machete to cut her as well, but she had already shouted for help and this saved her children:

*“…there is a time they caught me with poison I was about to place it in a cup… So, she came by, I do not know if its God that talked to her because I had the poison already and I wanted to kill them because they were innocently suffering by mainly lack of food … I bathed my children, because my plan was after giving them the poison they sleep, then I leave … I started also shouting and beating her, I looked for a machete to cut her, but she was faster than me, but she went out screaming (BHCIV 03).”*

##### Feelings of self-harm

It was also revealed that at times some participants felt the need of harming selves. One narrated that the thought of self-harm was always prompted by persistent negative thoughts of killing self of which she at times harmed herself:

*“Sometimes I feel like inflicting pain on myself, I feel like someone should slap me or pinch me so hard so that I stop having all these negative thoughts of killing myself, at a point I started slapping and pinching myself but it did not make me feel any better (MRRH 27).”*

#### Coping with postpartum depression

Four sub-themes were generated from the coming to terms with postpartum depression. These were spirituality (Christians, Muslims and Traditional African beliefs), termination of their marital relationships, acceptance, counselling and seeking medical treatment and finally perseverance.

Some mothers reported that they relied on spirituality, others resorted to crying and watching television, talking to a friend, health worker or relative for counseling or medical treatment. In addition, some mothers opted for attempting to leave their marital relationships and a few of them reported that they had terminated their marriages.

##### Spirituality: (Christians, Muslims and Traditional African beliefs)

The majority of participants revealed that they were relying on spirituality by engaging in prayers, participating in prayer groups, or regular attendance of church activities to overcome their challenges as a coping strategy. The majority of participants were Christians, and the minority were Muslims. Mothers taking part in the study shared their stories about how prayers had helped them overcome negative emotions like feelings of suicide and homicide. One mother voiced that her in-laws disliked her child to an extent that they always be-witched him by sending him evil spirits that almost killed him, which terribly stressed the mother. Traditional African spirituality was described through the use of “witchcraft” and traditional “witch doctors”. The role of spirituality in facilitating coping among mothers suffering from postpartum depression was clearly stated by:

*“… If it was not for God and his son Jesus Christ who died for us, I place all my troubles and he listens… surely I would not still be in this marriage. You see everything that comes I place to the Lord, I ask the Lord to see me through every situation that comes by in my family… (MRRH 13).”*

*“…You know; we the Muslims listen to Quran. During this terrible time, I engaged myself more in prayers and reading of the Quran as this makes me feel better…. Usually, when I was in a very bad mood, I would first cry, but quickly resort to praying or reading of the Quran which would relieve and I feel better (MRRH05).”*

*“There is a lot of witchcraft in this community and at one point, my in-laws sent some evil spirits and they affected my baby. My baby was very sick and almost died. My husband gave up on us, the situation was so stressful, and I was losing it until my neighbor came and advised me to look for a witch doctor who helped me to overcome the situation. Since then, I have learnt to consult witch doctors in several situations concerning my children, my health and my marriage. You know this witchcraft has actually helped me until now (MRRH 10).”*

##### Terminating their marital relationships

Mothers leaving their marital relationships was one of the alternatives available for overcoming the negative experiences in their postpartum period. As narrated, some participants said that it was better to quit and leave their partners and seek economic opportunities like working as a house help rather than keep suffering to the extent of wanting to kill themselves:

*“At first, I had thoughts of killing myself then I decided that instead of killing myself, I would rather leave this marriage. I would rather look for work as a house girl, earn some money that would take care of us, rather than keep suffering in his house to an extent of wanting to kill myself (MRRH 13).”*

*“I will try by all means to raise my child but if the situation becomes terrible, I think I will be forced to dump my baby somewhere and I just run away because I cannot accept to suffer with him... “we did not come together in this world (MRRH 15).”*

##### Acceptance, counselling and seeking medical treatment

Some mothers have received counselling services and expressed acknowledgement of what they were going through as one of the coping mechanisms, others have learned to accept this distressing condition. Others received medical pharmacological treatment for depression, insomnia and lack of energy to which they adhered and the situation improved as narrated:

*“I think it’s in my heart, I have a spirit of accepting and getting satisfied with what I have and what I don’t I let go… so counselling is the first kind of help in my own perspective (BHCIV 04).”*

*“… explained the situation to the health worker, like you right here, and he gave me some tablets to swallow to ease my pain and be able to sleep because sleep was hard to get …when they gave me those tablets, after swallowing them I would feel somehow relieved... woke up with energy. In case I fail to take them, I wake up without energy and then I realize that it’s that anger... (BHCIV 27).”*

Some mothers reported that the interface they had with the nurses in the health facility was not a good experience. They found it hard to go back and seek psychosocial treatment due to fear of being neglected.

*“I tried sharing my personal challenges with the nurses after birth, but they just ignored after blaming me for complaining too much. Some even talked badly to me, shouted and barked at me as if I was the cause of the problem. I was really stressed out since I could not get any help from my husband, family members nor the nurses from the hospital so I regretted ever sharing my problems with them (MRRH 15).”*

##### Perseverance

A few participants expressed the need to persevere with the hope that things will normalize 1 day. One of the participants who described perseverance was participant MRRH 04 who asserted that she was ready to stay in the marriage and face the challenges head on:

*“I love my husband very much… when a problem comes you have to face it head on, whether there is money or not… whether my husband abuses me or not… all this I just withstand and will not leave this marriage… these things of the husband abusing you and you pack things to leave, are not for me…I believe things will get better soon and we will be one happy family again (MRRH 04).”*

## Discussion

The study explored the lived experiences of Ugandan women who had recently recovered from a clinical diagnosis of postpartum depression. The five major themes that emerged coincided with the biopsychosocial framework of depression and these were: 1) somatic experiences (biological), 2) difficulties in home and family life (social), 3) negative emotions (psychological), 4) feelings of suicide, homicide and self-harm (psychological) and coping with postpartum depression. This section provides the discussion of the findings by juxtaposing the results of the present study with the existing literature.

### Somatic experiences of mothers diagnosed with PPD

Study participants reported somatic experiences which they attributed to the distress they were going through. Childbirth is associated with a range of physical and psychological effects on the mothers. For instance, in this study, several mothers reported sleep challenges characterized by interruptions in sleep patterns and sleepless nights during the postpartum period. The mothers attributed sleep problems to the care demands of the newborn and this is in tandem with previous studies [23, 24]. According to Walker and colleagues [25], the sleep deprivation and disturbance faced by mothers in the postpartum period affects memory, decision-making, psychomotor and mood. The revelations of mothers about sleep disturbance align with previous studies showing an association between sleep quality and postpartum depression [23–25].

Some participants reported unexplained headaches and body pain, with mother’s who delivered by caesarian section reporting headaches more often that those who delivered vaginally. Researchers from prior studies have reported increased headaches in women within 6 weeks after delivery [26]. Longitudinal research has explained that a drop in the hormonal levels, lack of sleep or dehydration experienced in the post-partum period contributes to headache occurrence and that women who deliver by cesarean section experience headaches more frequently than the women who had vaginal delivery [27]. This has been reported as a side effect of the epidural puncture following caesarian section.

In this study, mothers diagnosed with postpartum depression faced poor breast milk production. This is similar to what previous studies have reported that mothers with PPD were more likely to stop breastfeeding in the early months after birth [28–30]. This may be attributed to PPD often forcing mothers to resort to non-optimal feeding practices such as premature bottle feeding of infants and early weaning. Prior research demonstrated that compared to mothers who were not depressed, mothers with PPD exhibit low breastfeeding confidence, low levels of breastfeeding, self-efficacy and lack of satisfaction of their infant feeding methods [31, 32] which is related to the distress.

Mothers with postpartum depression from the present sample experienced loss of appetite for food which may have led to weight loss. According to the revelations of the participants, there were disruptions in feeding habits due to wariness, disturbance because of child crying a lot and increased workload related to childbirth. To the contrary, some studies have reported weight gain among mothers suffering from PPD and this has been explained by depression that leads to changes in sleeping patterns, engagement in physical activity, and changes in dietary intake that may increase the risk of weight retention or gain [33]. Lox and Treasure [34] reported that high stress levels and major life disruptions experienced by mothers with PPD impede physical activity leading to weight gain.

Mothers reported that they sometimes lacked energy due to the distress to an extent that they could not cultivate land to provide their own food, which is their source of income, or even lacked energy to eat even when there was food. There is already evidence provided by existing literature that fatigue is a common occurrence among mothers with PPD [35]. The prevalence of fatigue and related symptoms reduces self-care behaviors, participation in pleasurable activities among mothers and promotes persistent low moods. This also affects the mother’s care for the infant leading to poor childcare practices [36]. Findings of this study affirm this claim that low energy and fatigue are a major factor among mothers in southwestern Uganda who are diagnosed with PPD.

### Difficulties in home and family life

The second prominent theme was difficulties in home and family life which coincides with the social experiences of mothers diagnosed with PPD in the biopsychosocial model of depression.

Mothers in the current study sample reported feeling overwhelmed with domestic chores. Motherhood is often associated with increased time and demand for childcare and housework such as breastfeeding, washing the clothes and preparing for the baby which are additional tasks to the chores a woman is expected to perform on a daily basis. Sleep disturbance is also common among mothers during the postpartum period instigated by the baby crying at night or breastfeeding. Such experiences limit opportunities for the couples to share leisure activities and intimacy leading to stressful feelings. It is against this background that studies have linked increased housework and PPD [37, 38]. Therefore, specific interventions that reduce domestic chores in low-income countries settings such as water harvesting and energy saving technologies are critical in reducing stressful experiences of these mothers thus mitigating the increasing incidence of PPD.

The experience of lack of social support from other family members was also reported by mothers that participated in the study. Ugandan settings are often characterized by extended families and support is expected from other family members including parents, siblings, in-laws and close family friends. Family members are expected to provide social support such as accompanying the mother to the health facility, babysitting, helping with domestic chores, providing guidance regarding motherhood and childcare while the mother rests. In situations where social support from partner is already lacking, expectation for support from others especially family members, relatives and close friends are high. Lack of such social support is likely to invoke disappointment, anxiety, feelings of neglect and other forms of psychological distress among mothers in the postpartum period. Previous studies have also found that lack of social support was associated with PPD [39]. According to Yamada [39], there are several scenarios that can explain the linkage between mothers’ lack of support from others and PPD. The first scenario is the stress resulting from the mother comparing herself with other mothers who receive support. Mothers with support can share their challenges related to motherhood and childcare and receive guidance, encouragement and comfort; this may not be same for mothers without social support from others. This implies that partner support alone is not adequate, but new mothers need supplementary efforts from others in their family and community.

In addition, the experience of discord at home was reported by mothers in the study sample. These experiences manifested through verbal, emotional and sometimes physical abuse by the majority of participants. Mothers further reported that fighting was common if the partner was not in position to support the mother financially and morally. Alcohol abuse and being HIV/AIDS positive were also linked to this type of intimate partner violence (IPV). In a country like Uganda where gender based violence is a common occurrence, it is not surprising that incidents of IPV were frequently during the study [40]. Participants in the present study characterized IPV as aggressive behavior with various types of abuse such as verbal attacks, physical assault, sexual violence, neglect and victimization. The findings of the present study are in tandem with observations of other studies from Africa and other low-income countries [10, 41]. In a number of studies, alcoholism and substance abuse has been reported as a key trigger of IPV during the postpartum period [11, 41].

Mothers in this study experienced financial constraints. They reported constant concerns about meeting the costs of giving birth and afterbirth care as a result of limited sources of income, loss of jobs and refusal/failure by spouses to provide the required financial assistance. The majority of participants in the study revealed how they faced financial and other logistical constraints such as food, housing and clothing for their children. This shows that financial and economic stress is an important experience of PPD that women had as mothers reported it to be a key challenge in the postpartum period. During this period, there is increased financial stress to raise the infant and childbearing comes with extra financial demands. Prior systematic review of literature has reported that women with financial needs were more prone to suffering from PPD [42].

The COVID-19 pandemic appears to have worsened the financial challenges faced by women diagnosed with PPD. This pandemic resulted in loss of jobs and business opportunities due to various restrictions, an abrupt national lock down, closure of small scale and micro businesses like roadside food stalls and vending that often provided women with livelihood opportunities. Due to such restrictions, many mothers or their partners lost their jobs. The restrictions made it difficult to access basic needs such as food and healthcare. Studies have reported that many women faced extreme fear and stress due to the challenges caused by COVID-19 pandemic [43].

### Negative emotions

Anger, self-blame, despondency and feelings of loneliness and regrets about conceiving or marriage emerged from this theme. Several forms of emotional distress were attributed to spouses refusing to take responsibility during pregnancy and in the postpartum period, financial difficulties to care for health costs associated with childbirth, spouse not being happy with the sex of the baby and unintended pregnancies. Unintended pregnancies have particularly been reported in previous studies as a key predictor of postpartum depression and its associated experiences such as feeling of regret, self-blame and anger [44–46]. A study done in Ethiopia reported that mothers who had unwanted pregnancies were two times more likely to be depressed than women whose pregnancies were planned [46].

Despondency and uncertainty were common postpartum experiences reported by these mothers and it was largely attributed to strained relationships with the participants’ partners. Most participants reported being in strained relationships, some reported being involved in casual sexual relationships, while others defined their relationship as being engaged with a partner who had a stable relationship elsewhere and others could not clearly describe their relationship with their partners. This suggests that the claim that the quality of partner relationship was a lived experience of PPD as has been reported by a number of studies [38, 47]. It has been reported that mothers who perceived their partners support as being less than their expectations were more likely to experience greater severity of postpartum depression symptoms [48]. Burke [49] has also demonstrated a relationship between disharmony, partner incompatibility and marital conflicts and symptoms of PPD. The causes of disharmony vary and may include unintended pregnancies, casual relationships that may not meet the support needs of the mothers, the baby not being the preferred sex of the partner and drug abuse. Giordano and colleagues [50] reported that poor communication including verbal interactions and not knowing what to say to a partner influenced depressive symptoms among mothers.

### Feelings of suicide, homicide and self-harm

In this study, participants reported experiences of suicidal ideation or attempts during the postpartum period which calls for urgent public health intervention. These suicidal thoughts were attributed to various negative experiences including abuse by partners, lack of social support from partner or/and others and financial constraints. The experience of suicidal thoughts has been reported by previous studies [7]. Some studies have reported that these thoughts may be influenced by other factors not directly related to the postpartum experiences such as history of self-harm or suicidal attempts, a family history of suicide, increased levels of hopelessness, childhood physical or sexual abuse and neglect [7, 8]. Demographic factors such as being unmarried, low economic status and child pregnancy have also been documented to increase risk of suicidal thoughts among postpartum depressed mothers [51].

The findings of this study further showed that experience of having PPD included homicidal thoughts. Homicidal thoughts mean a situation where someone idealizes or feels like killing another person. In the context of this study, some mothers experienced feelings of wanting to kill their infants (infanticide), partners or the older children. Infants are generally the leading victims of homicides related to PPD [52]. Various studies have reported that homicidal thoughts and actual cases of homicides were common among postpartum depressed mothers caused by the feeling of neglect and abuse, rejection of the newborn by the partner and in some cases as a revenge on the infant’s father [53, 54]. Study participants also reported the thought to kill their partners using varying methods such as poisoning or using objects such as machetes. Such results show how PPD is a serious risk not only to the wellbeing of the mother but also to their infants, their partners and other close relatives, thus indicating the need for substantive response to this issue.

According to this study thoughts of self-harm were prompted by persistent negative thoughts. These results are in line with related studies which noted that experiences of self-harm were common among postpartum depressed mothers especially young women, single and divorced mothers, mothers who lack social support, and mothers who are disadvantaged economically [55]. The feelings of self-harm have the potential to increase the risk of suicidal thoughts and actual suicides if untreated.

### Coping with postpartum depression

This study documents various coping strategies used by mothers diagnosed with PPD. Most of the mothers interviewed relied on spirituality, including Christian, Islam and traditional African beliefs, as their source of strength to cope. Mothers revealed how they engaged in prayers as individuals, through prayer groups and participating in church activities to cope with negative experiences caused by PPD. Various studies have reported spirituality and religious practices as a major coping strategy [55, 56]. This is in line with a study that was carried out in Ethiopia which reported that most mothers opted to affiliate themselves with religion as a way of coping with stressful experiences [57].

Some mothers diagnosed with PPD sought counseling and medical treatment, but most did not seek help for the depressive symptoms. This is in line with previous studies which have shown that the number of mothers with PPD seeking professional counseling and medical treatment is low and that the majority prefer to receive counseling from religious leaders in the communities, family friends and relatives [58, 59].

Some mothers opted to quit their intimate relationships, especially those who felt the quality of the relationship was responsible for their distress. Quitting relationships was considered as a choice because most mothers suffering from PPD blamed the relationship quality for their challenges. Related studies have reported that most depressed mothers related their relationships with partners as distant, cold and difficult [60]. Another study reported that breakups were more common among depressed mothers compared to the non-depressed mothers [61].

### Strength and limitations

The main strength of this study is that the research team included 30 mothers diagnosed with PPD from diverse age groups, educational backgrounds, economic status, marital status and rural or urban settlements. The sample size was adequate for qualitative studies and the diversity of participants makes the study representative. However, the research team acknowledges that being a qualitative study, drawing causal conclusions is untenable. However, the study juxtaposed the narratives of the participants and their background information to make inferences. Another limitation is that this study only targeted mothers who had recovered from PPD and therefore findings may not be representative of women with ongoing or persistent depression.

### Implications for practice and research

The documentation of these experiences is significant in providing clear understanding of PPD and informing healthcare providers the lived experiences of these mothers.

Effective assessment of PPD clinical presentations of the psychosocial, biological experiences and negative coping strategies must be incorporated into the postpartum care package for all postpartum mothers. Understanding the subjective experience will enable nurses and health workers to identify women at risk of developing PPD and establish effective interventions for distressed women during the postpartum period.

The findings of the study showed that poor relationship quality and lack of partner support aggravated negative experiences of mothers diagnosed with PPD. This highlights the importance of partner involvement in postpartum services is critical to increase the opportunities for stronger and more positive family and relationship bonding during this period. Partner involvement could potentially also reduce intimate partner violence.

Unintended pregnancies were reported as a prime experience by women who had PPD. A longitudinal study exploring the link between PPD and unintended pregnancies in this population is recommended to mitigate PPD and its after effects.

The local health districts should increase the availability of mental health services to support women with PPD at the local health facilities. This will promote mental health care including early identification and management of PPD and mitigate the negative experiences lived by these mothers. The nurses and midwives working in the postnatal clinics should implement a validated screening and diagnostic process to identify women suffering from PPD. In addition to augmenting the limited present counselling services currently provided in the postnatal clinics, the clinic-based nurses and midwives should leverage the benefits described by the study participants from spiritual coping and refer the identified mothers to take advantage of their existing spiritual beliefs to maximize recovery. The nurses and midwives should involve local community health workers and the local spiritual leaders from churches, mosques and traditional African healing clinics by sensitizing and educating them about PPD. This can increase their encouragement and engagement of new mothers to enhance these spiritual coping strategies as highlighted by the study participants.

Future research may explore postpartum depression in men to better understand men’s experience in the postpartum period and longitudinal studies establishing the long term effects of postpartum depression.

## Conclusion

Five major themes that emerged were somatic experiences, difficulties in home and family life, negative emotions, feelings of suicide, homicide and self-harm and coping with postpartum depression. Suicidal and homicidal thoughts are important parts of the postpartum depression experience, and may put the lives of the mothers, their spouses and babies at a great risk. Poor relationship quality, intimate partner violence and lack of financial resources contribute significantly to the negative emotional experiences of mothers with PPD. It is evident that mothers suffering from postpartum depression go through varying psychological, social and biological experiences.

## Supplementary Information

**Additional file 1.** Interview Guide.

## Data Availability

The data that support the findings of this study are available from the first and corresponding author, Catherine Atuhaire but restrictions apply under license for the current study.

## Abbreviations

BHC IV: Bwizibwera Health Centre IV
DSM-5: Diagnostic and Statistical Manual of Mental Disorders, 5th Edition
IDI: Indepth interviews
IPV: Intimate partner violence
KHC IV: Kinoni Health Centre IV
PPD: Postpartum Depression
MCH: Maternal and child health
MRRH: Mbarara Regional Referral Hospital
SSA: Sub-Saharan Africa
