# Supplementary material for: Lived experiences of Ugandan women who had recovered from a clinical diagnosis of postpartum depression: a phenomenological study
Source: BMC Pregnancy Childbirth. 2021 Dec 13;21:826. doi: 10.1186/s12884-021-04287-2 (PMC8666838; doi:10.1186/s12884-021-04287-2)
Supplement: Supplementary file 1 — Additional file 1. Interview Guide. [file 12884_2021_4287_MOESM1_ESM.docx]

**INTERVIEW GUIDE FOR DOCUMENTING THE LIVED EXPERIENCES OF UGANDAN MOTHERS CLINICALLY DIAGNOSED WITH POSTPARTUM DEPRESSION**

Study Number: ________________________________

Date of Interview: ______________________________

Health facility: _________________________________

Date of Birth: __________________________________

Date of Delivery: _______________________________

**Interview Questions**

1. Please can you tell me how your experience has been from the time you gave birth to to-date?
2. Was there anything that made your experience difficult? If so, what?
3. How have you been trying to deal with such feelings or experience?
4. How has the condition affected the way you care for your baby?
5. How have the people around you (husband, close relatives and friends) responded to your condition?

?

1. What has been your experience with the health workers in trying to address this condition?
2. What would you have changed about this experience if you could?

**Probing Questions**

1. If you have given birth before, do you find this experience is new to you?
2. Was there any suffering that was beyond you?
3. Was there anything else that would have helped during this period? If so, what?
4. Any marital or child related beliefs to PPD?
5. Stigma and discrimination experience from partner, relatives and friends will be probed for.

Thank you for your cooperation
